# Supplementary material for: Misfolded amyloid-β-42 impairs the endosomal–lysosomal pathway
Source: Cell Mol Life Sci. 2020 Feb 5;77(23):5031–43. doi: 10.1007/s00018-020-03464-4 (PMC7658065; doi:10.1007/s00018-020-03464-4)
Supplement: Supplementary file 1 — Supplementary file1 (DOCX 2835 kb) [file 18_2020_3464_MOESM1_ESM.docx]

**SUPPLEMENTARY INFORMATION**


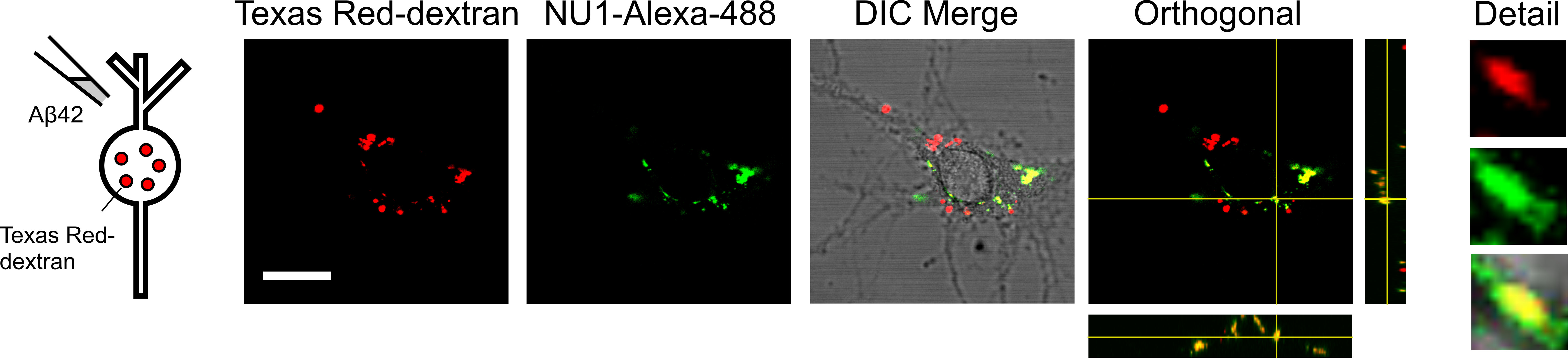


**Figure S1. Immunolabelling of internalised 10 μM Aβ42 oligomers and lysosomes labeled with Texas Red-Dextran.** Co-labelling with Texas Red-Dextran to label lysosomes and anti-oligomer antibody NU1 to label internalized Aβ42 oligomers shows that oligomers co-localise to lysosomes as shows by overlapping red and green signals in x, y and z. Scale bar is 10 μm.

*
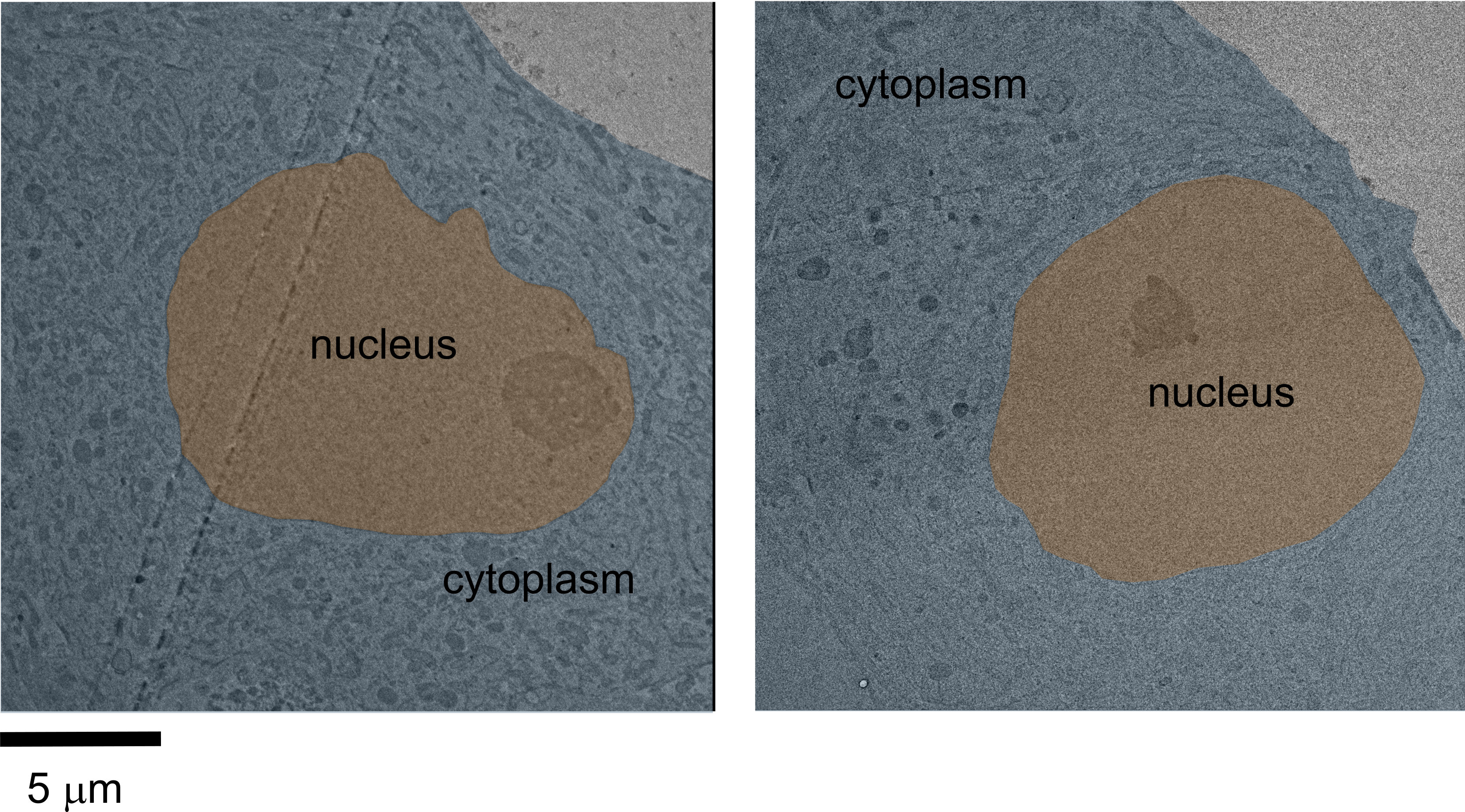
*

**Figure S2. Aligned and overlaid transmission electron microscopy images in a buffer-treated neuron.** Incubation was for 14 days with volume equivalent to 5 μM Aβ42 oligomer-treatment condition.

*
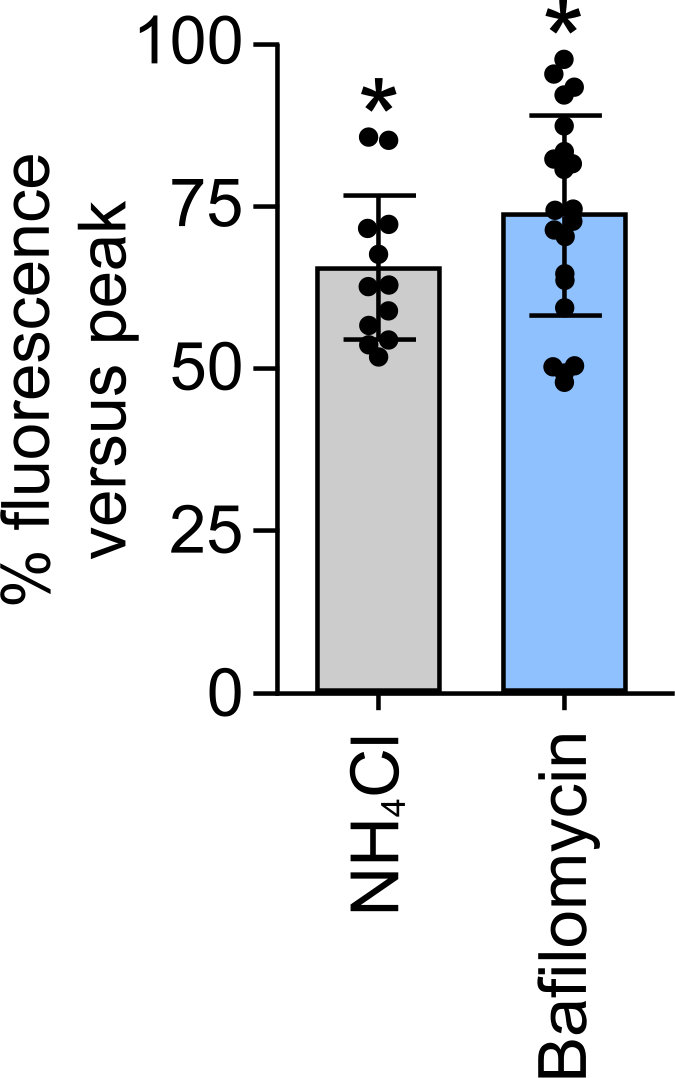
*

**Figure S3. Mean CypHer fluorescence intensity within the neuronal cell body cytoplasm increases following addition of Ova-Cy and is reduced after addition of NH_4_Cl or bafilomycin.** Reduction of mean fluorescence intensity after 10 minutes (NH_4_Cl) or 30 minutes (bafilomycin) compared with maximum intensity reached. Following addition of NH_4_Cl, the fluorescence intensity drops by 34.5 % ± 11.6 % within 10 minutes, compared to the maximum (SD, three experiments, 12 cells, p=0.0004 (***)). Bafilomycin had a similar effect, reducing the fluorescence intensity by 26.3 % ± 15.8 % from the maximum after 30 minutes (SD, three experiments, 21 cells, p=0.03 (*), unpaired t-test).

*
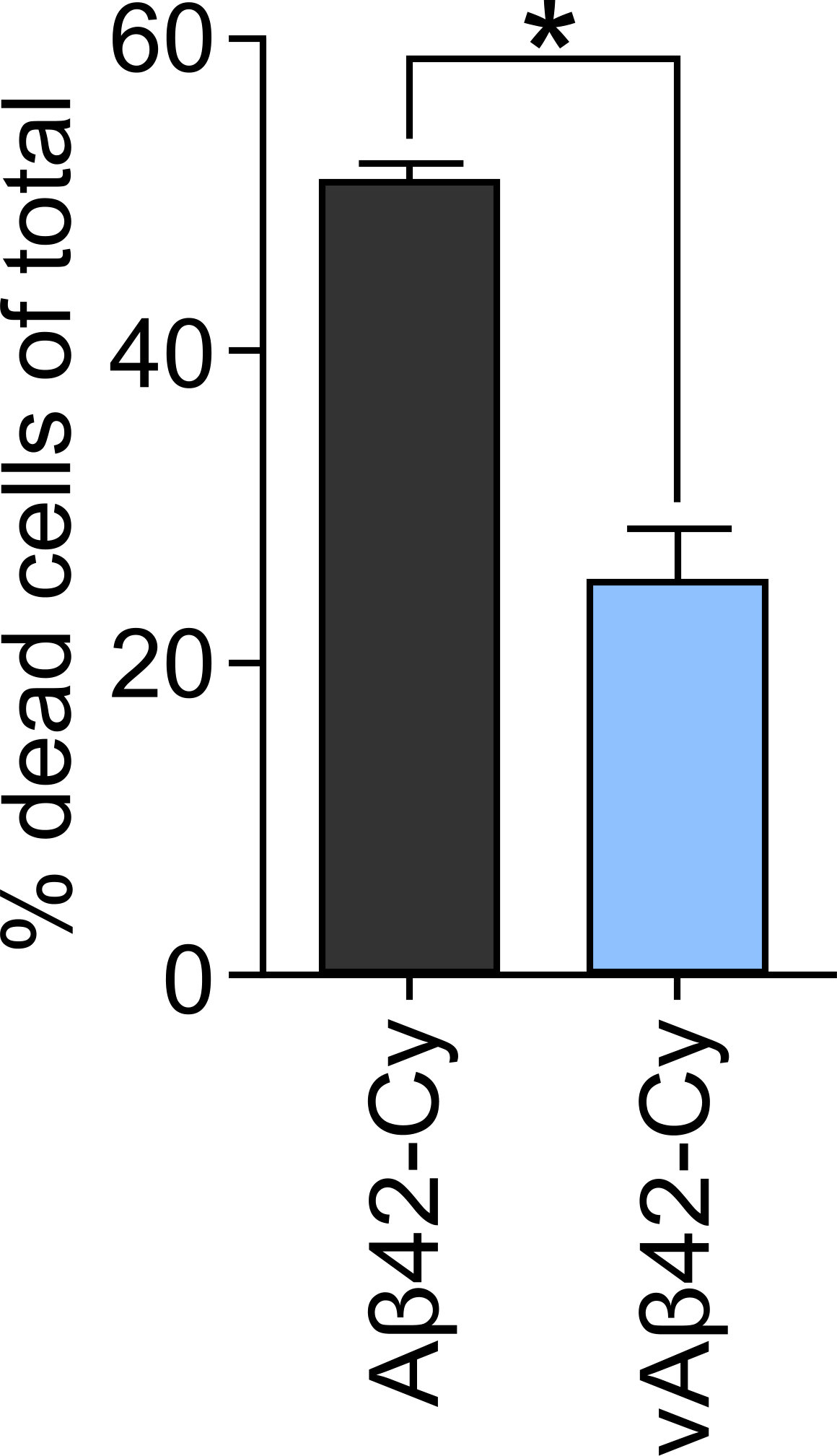
*

**Figure S4. Toxicity of 10 μM Aβ42-Cy and vAβ42-Cy to primary hippocampal neurons following 72 h incubation assessed using the Readyprobes live/dead assay**. 8-10 FOV were used per experiment, n (cells) Aβ42-Cy = 153 (78 dead, 51.0%), vAβ42-Cy = 169 (43 dead, 25.4%). t-test, unpaired with Welch’s correction, p = 0.0035.

*
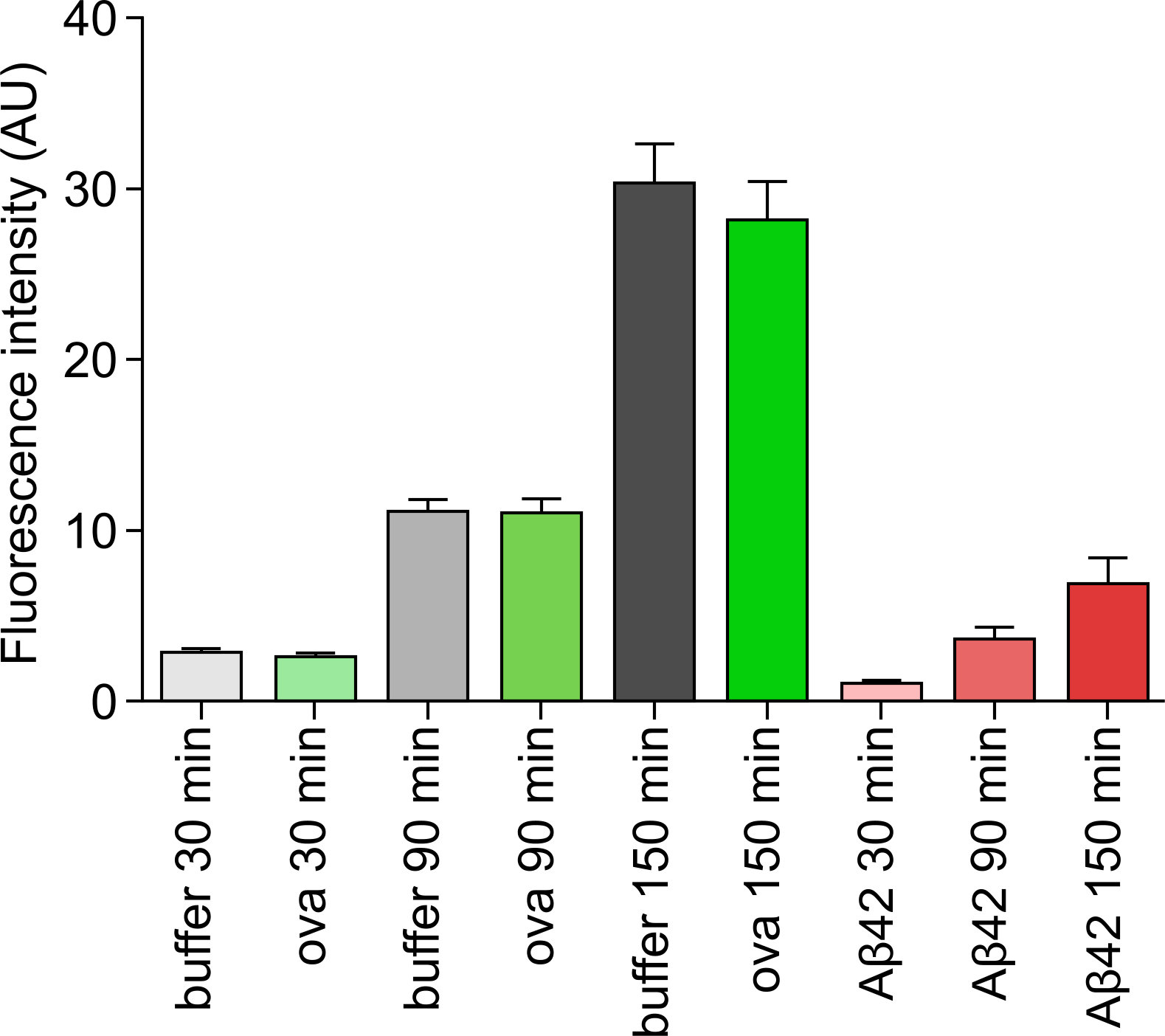
*

**Figure S5. Fluorescence intensity arising from Ova-Cy in neuronal cell bodies in cells either treated with 10 μM ovalbumin or buffer.** Both ovalbumin and buffer treatment (equivalent volumes) were for 72 h and imaged at 30, 90 or 150 minutes. Mann-Whitney unpaired t-test showed no significant difference between ovalbumin or buffer treated cells at any time point (p > 0.5).
